# Safety Assessment of *Bacillus subtilis* MB40 for Use in Foods and Dietary Supplements

**DOI:** 10.3390/nu13030733

**Published:** 2021-02-25

**Authors:** Jessica L. Spears, Richard Kramer, Andrey I. Nikiforov, Marisa O. Rihner, Elizabeth A. Lambert

**Affiliations:** 1 BIO-CAT Microbials LLC, Shakopee, MN 55379, USA; rkramer@bio-cat.com; 2 Toxicology Regulatory Services, Charlottesville, VA 22911, USA; ANikiforov@ToxRegServ.com (A.I.N.); MRihner@toxregserv.com (M.O.R.); 3 Charles River Laboratories Ashland LLC, Ashland, OH 44805, USA; Liz.Lambert@crl.com

**Keywords:** *Bacillus subtilis* MB40, probiotics, safety, tolerability

## Abstract

With the growing popularity of probiotics in dietary supplements, foods, and beverages, it is important to substantiate not only the health benefits and efficacy of unique strains but also safety. In the interest of consumer safety and product transparency, strain identification should include whole-genome sequencing and safety assessment should include genotypic and phenotypic studies. *Bacillus subtilis* MB40, a unique strain marketed for use in dietary supplements, and food and beverage, was assessed for safety and tolerability across in silico, in vitro, and in vivo studies. MB40 was assessed for the absence of undesirable genetic elements encoding toxins and mobile antibiotic resistance. Tolerability was assessed in both rats and healthy human volunteers. In silico and in vitro testing confirmed the absence of enterotoxin and mobile antibiotic resistance genes of safety concern to humans. In rats, the no-observed-adverse-effect level (NOAEL) for MB40 after repeated oral administration for 14 days was determined to be 2000 mg/kg bw/day (equivalent to 3.7 × 10^11^ CFU/kg bw/day). In a 28 day human tolerability trial, 10 × 10^9^ CFU/day of MB40 was well tolerated. Based on genome sequencing, strain characterization, screening for undesirable attributes and evidence of safety by appropriately designed safety evaluation studies in rats and humans, *Bacillus subtilis* MB40 does not pose any human health concerns under the conditions tested.

## 1. Introduction

Although the effects of microorganisms on the human body have been studied for well over a century [1], research specifically on microbes of the human gut has intensified over the past decade. This research has led to the discovery of commensal and beneficial bacterial species that support healthy gastrointestinal physiology. In addition, oral supplementation with probiotics has become increasingly popular. Probiotics are live microorganisms that, when administered in adequate amounts, confer a health benefit on the host [2]. This globally recognized definition sets a critical requirement that probiotics, such as specific strains of *Lactobacillus* spp., *Bifidobacterium* spp., and *Bacillus* spp., demonstrate a health benefit in a properly designed clinical study. Examples of substantiated health benefits of probiotics include the support of gastrointestinal health, immune health, and promotion of the growth of beneficial gut bacteria [3,4]. More specifically, probiotics such as *Bacillus coagulans* and *Bacillus subtilis* have been clinically shown to improve dietary protein digestion [5] and help with occasional gas and bloating [6,7]. Despite the growing number of benefits associated with oral probiotic use, strain-specific safety is, first and foremost, a priority.

As natural inhabitants of the human gut, many strains of *Lactobacillus* spp. and *Bifidobacterium* spp. have an established history of safe use in dietary supplements and foods such as yogurt, kefir, and cheese. While spore-forming *Bacillus* species have traditionally been described as soil-borne bacteria, they too have been described in the naturally occurring human gut microbiota, albeit less represented in commercially available probiotic products. Multiple independent studies have reported the presence of *Bacillus* spp., and specifically *Bacillus subtilis*, in intestinal and human fecal samples, independent of any probiotic supplementation. Collectively, these data show that *Bacillus* spp. occurs in the human gut in large enough numbers to be a resident gut commensal bacterial species [8,9,10]. 

Additionally, *Bacillus subtilis* has been safely used in traditional fermented foods of many east Asian cultures for centuries. Producers of natto, a traditional Japanese fermented soybean food, have utilized *Bacillus subtilis var. natto* for commercial production since the early 1900s [11]. *Bacillus subtilis* strains are naturally present in Korean kimchi, Egyptian kishk, and in a variety of cultural adaptations of fermented soy including miso and thua nao [12,13,14,15]. Sequencing and characterization of these strains support the safe use of *Bacillus subtilis* in foods and dietary supplements [16,17]. Safe use of *Bacillus subtilis* strains is supported not only in healthy adults [18,19] but also in pediatric populations [20]. 

However, not all strains of a particular species should be assumed safe for use in dietary supplements and food despite species inclusion on published lists of organisms generally presumed safe. The European Food Safety Authority, for example, publishes a qualified presumption of safety (QPS) list of biological agents which includes *Bacillus subtilis* [21]. While many *Bacillus subtilis* strains are safe for consumption, some strains of *Bacillus subtilis* have been shown to produce the hemolytic enterotoxin Hbl [22], making such strains unsuitable for use in foods and supplements. General assumptions at the species level are helpful to inform deeper review and precise scientific opinion for specific applications. However, each specific strain must be scrutinized for safety and tolerability similar to the requirement to demonstrate the efficacy of specific strains.

This work aims to determine the safety and tolerability of a unique strain of *Bacillus subtilis* subsp. *subtilis* MB40 for human consumption. In 2015, Pariza et.al. [23] put forth a list of critical questions in the form of a decision tree to consider for determining the safety of a specific microbial strain for human and animal consumption. Utilizing this decision tree, numerous in silico, in vitro, and in vivo studies were used to determine whether *Bacillus subtilis* MB40 poses any safety concerns to humans. These studies included whole-genome sequencing and data analysis, screening for enterotoxin production and antibiotic susceptibility, repeated-dose toxicity testing via dietary administration in a rodent model, and a human safety and tolerability study to thoroughly evaluate and demonstrate the safety of *Bacillus subtilis* MB40.

## 2. Materials and Methods

### 2.1. Test Article

*Bacillus subtilis* MB40 (ATCC Accession No. PTA-122264, hereafter referred to as “MB40”) is a Gram-positive, spore-forming facultative bacterium. MB40 is grown under aerobic conditions to its spore form and spray dried on site at BIO-CAT Microbials (Shakopee, MN, USA). Pure MB40 spray-dried spores blended with a suitable diluent (e.g., maltodextrin) or pure liquid cultures were used in all in vitro and in vivo studies at reported concentrations. All in silico studies were completed using genomic DNA extracted from pure MB40 culture. Due to the nature of various assays, different strain concentrations were used as necessary. Concentrations are noted per individual study.

### 2.2. Enterotoxin Screen (In Silico)

A nucleotide BLAST^®^ search was completed via the NCBI website (http://blast.ncbi.nlm.nih.gov/Blast.cgi (accessed on 23 December 2020)) to determine the presence or the absence of toxin genes commonly associated with *Bacillus* spp., especially *B. cereus*. First, positive control genes were identified: *B. subtilis* glutamyl-tRNA(Gln) amidotransferase subunit (gatA) and *B. cereus* methionyl-tRNA synthetase (metG). These genes were used as a query against the subject sequence MB40 genome to demonstrate the chosen nBLAST algorithm was able to generate a match when one existed. Second, each toxin DNA sequence was identified using NCBI gene (http://www.ncbi.nlm.nih.gov/gene (accessed on 23 December 2020)). Finally, each toxin gene DNA sequence was used as a query against the MB40 genome sequence. All nucleotide BLAST alignments were performed using a discontinuous megablast with default algorithm parameters.

Additionally, virtual polymerase chain reaction (PCR) (accessed at http://insilico.ehu.eus/user_seqs/PCR (accessed on 23 December 2020)) was used to search the *B. subtilis* MB40 genome for toxins via gene primer matches [24]. Ten sets of sequence primers (primers listed in Table S1) for toxin DNA amplification were identified through primary literature sources and used to complete the virtual PCR [25,26,27]. The following parameters were used to closely mimic an actual PCR run: 2 mismatches allowed, no mismatch allowed in the last nucleotide of the 3′ end, and a maximum band length of 10,000 nucleotides. As a positive control for the primers, the same set of primers were run against the *B. cereus* genome, generating matches in all cases. As a control for the virtual PCR protocol for “across-species” matches, primers for a sporulation gene (spoIVA) were used to search both the MB40 and *B. cereus* genome, finding matches in both cases. Primers for spoIVA and 16S RNA were used to show that the program would find a match in the MB40 genome when one was present.

### 2.3. Enterotoxin Screen (In Vitro)

The absence of major enterotoxins in MB40 was confirmed using kits commercially available at the time of study: 3M Tecra™ (St. Paul, MN, USA) and Oxoid™ BCET-RPLA (Hampshire, UK). The 3M Tecra™ kit is able to detect *Bacillus* diarrheal enterotoxin (BDE) at or above a concentration of 1 ng/mL. The Oxoid kit is able to detect *B. cereus* enterotoxins (BceT) at a concentration at or above 2 ng/mL. Each kit included internal positive and negative controls that were run alongside each trial. 

A frozen stock vial of MB40 was used to streak a trypticase soy agar (TSA) plate for isolation. TSA plates were incubated overnight at 35 ± 2 °C and well-isolated colonies were used to inoculate 100 mL of MB40 growth medium. After 16–20 h of growth at 35 ± 2 °C with shaking, a 1 mL sample was harvested from each flask; the cells were separated from the spent medium via centrifugation. The assays were completed and data analyzed according to the manufacturer’s protocol. Three trials were run independently of each other and all data were independently interpreted by at least two researchers. All interpretations were completed in a single-blinded manner such that the researcher interpreting the data did not know which samples were controls or samples. All data reported are an average of at least three trials.

### 2.4. Antibiotic Sensitivity Testing

The CLSI Disc diffusion reference method, which is based on validated Kirby–Bauer methods, was utilized following published guidelines established by the Clinical Laboratory Standards Institute (CLSI) subcommittee on Antimicrobial Susceptibility Testing [28].

A frozen stock vial of MB40 was used to streak a TSA plate for isolation. TSA plates were incubated for 18–24 h at 35 ± 2 °C. At least three typical, well-isolated MB40 colonies were suspended in Butterfield’s Buffer to a 0.5 McFarland standard equivalent (Hardy Diagnostics; Santa Maria, CA, USA).

The standardized inoculum was used to inoculate room-temperature, 100 mm Mueller–Hinton Agar (MHA) plates (Hardy diagnostics) by covering the entire plate as described by the CLSI. BBL™ Sensi-Disc™ discs containing the CLSI-prescribed amount of antibiotic (Becton, Dickinson and Company; Franklin Lakes, NJ, USA) were loaded into a BBL™ Sensi-Disc™ dispenser and dispensed onto each inoculated plate within 15 minutes of inoculation. Four discs were evenly placed on each 100 mm plate. Plates were then inverted and incubated for 16–18 h in ambient air at 35 °C.

Inhibition zones were detected and measured to the nearest millimeter using a ProtoCOL 2 automatic colony counting and zone measuring instrument with ProtoCOL 3 software (Synbiosis, Frederick, MD, USA). Zone measurements were visually inspected and confirmed. If no zone was present, then 6 mm, the diameter of the disc, was recorded. *Escherichia coli* ATCC 25922 and *Staphylococcus aureus* ATCC 25923 were used as quality control test organisms according to CLSI protocols. All tests were performed in triplicate and all zone measurements reported are an average of those three trials.

### 2.5. Minimum Inhibitory Concentration

MB40 was evaluated against 8 antibiotics for in vitro antimicrobial activity (BioSciences, Bozeman, MT, USA; report number 1808366-202). The Minimum Inhibitory Concentration (MIC) of each antibiotic was determined based upon the methodology described in CLSI Document M07-A10 [29]. The concentration of MB40 cells used per well was 7.50 × 10^5^ CFU/mL. *Enterococcus faecalis* (ATCC Accession No. 29212) and *Staphylococcus aureus* (ATCC #29213) (1.32 × 10^6^ and 2.40 × 10^6^ CFU/mL, respectively) were tested in tandem with MB40 in order to verify the methodology performed in this study, and exhibited MICs within the CLSI quality control range. Ten different dilutions of each antibiotic were tested to determine each MIC.

### 2.6. Oral Toxicity Study in Rats

A rat 14 day oral toxicity study was conducted at Charles River Laboratories (CRL) Ashland, LLC (formerly WIL Research; Ashland, OH, USA) during September to October 2015 (study number WIL-274501). The study protocol was designed in general accordance with FDA Redbook 2000 Testing Guideline IV.C.3.a, Short-Term Toxicity Studies with Rodents [30], with the exception that collected tissues were preserved but not examined microscopically (study design schematic shown in Figure S1). 

Male (*n* = 45) and female (*n* = 45) Sprague–Dawley [Crl:CD(SD)] rats were obtained from Charles River Laboratories, Inc. (Raleigh, NC, USA) and were approximately 5.5 weeks old upon receipt. All animals were housed for a 12 day acclimation period and were observed twice daily for mortality and changes in appearance or behavior. All animals were individually housed in an environmentally controlled room (temperature 22 ± 3 °C and relative humidity 50 ± 20%) with a 12 h light/dark cycle. Animals were maintained in accordance with the Guide for the Care and Use of Laboratory Animals [31] in a facility accredited by the Association for Assessment and Accreditation of Laboratory Animal Care (AAALAC) International.

Animals received PMI Nutrition International, LLC, Certified Rodent LabDiet^®^ 5002 (meal) and reverse osmosis-treated drinking water ad libitum throughout this study. The animal feed and drinking water were determined to be free of contaminants at concentrations that would interfere with the objectives of this study.

After acclimation, all animals suitable for assignment to this study were assigned 1 of 4 test groups using a computerized randomized procedure based on body weight stratification in a block design. Individual body weights were within ±20% of the mean for each sex. Each group consisted of 10 males and 10 females and were approximately 7 weeks old at first dose. Individual body weights ranged from 201 to 249 g for males and from 153 to 191 g for females.

The test article, MB40, was supplied as a spray-dried spore preparation (blended with maltodextrin) with an activity level of 1.85 × 10^11^ CFU/g. Dose formulations were prepared daily at concentrations of 50, 100, and 200 mg/mL (0.925, 1.85, and 3.7 × 10^10^ CFU/mL, respectively) in deionized water. Test article doses of 500, 1000, and 2000 mg/kg bw/day were administered to groups of 10 male and 10 female rats by oral gavage in a dose volume of 10 mL/kg. The doses were equivalent to 9.25 × 10^10^, 1.85 × 10^11^ and 3.7 × 10^11^ CFU/kg bw/day. Based on average initial body weights, the doses in terms of CFU/day were 2.18 × 10^10^, 4.33 × 10^10^, and 8.51 × 10^10^ (males) and 1.71 × 10^10^, 3.38 × 10^10^, and 6.84 × 10^10^ (females). A vehicle control group was concurrently administered deionized water on the same daily dosing regimen as the test article-treated groups. 

Homogeneity and concentration of the test article formulations were confirmed prior to the initiation of dosing and at appropriate intervals during this study. The activity (CFU/mL) of the analyzed formulation samples was within the laboratory’s Standard Operating Procedure-defined acceptance criteria (i.e., 85% to 115% of target). 

Animals were evaluated twice daily for mortality and moribundity. Clinical examinations were performed daily at the time of dose administration and 1–2 h following dose administration. Detailed physical examinations were performed weekly beginning during the acclimation phase prior to initiation of dose administration. Individual body weights and food consumption were recorded weekly. Final body weights (fasted) were recorded on the day of the scheduled necropsy, i.e., study Day 15. 

Clinical pathology evaluations (hematology, coagulation, serum chemistry, and urinalysis) were performed on all rats at the scheduled termination. The animals were fasted overnight prior to blood collection while in metabolism cages for urine collection. Complete necropsies with gross pathological examinations were conducted on all animals and organ weights were measured for a preselected list of organs. A standard listing of tissues and organs were examined macroscopically from all animals. 

Statistical analysis was performed for body weight, body weight change, food consumption, clinical pathology, and organ weight data using a parametric one-way analysis of variance (ANOVA) [32] to determine intergroup differences. Normality and homogeneity of variance of the data were verified. If the ANOVA revealed statistically significant (*p* < 0.05) intergroup variance, Dunnett’s test [33] was used to compare the test article-treated groups to the control group.

### 2.7. Human Clinical Safety and Tolerability Trial (Single-Blind Design)

A single-blinded, placebo lead-in clinical study of 30 healthy adult participants was conducted to determine the tolerability of MB40. This study was conducted at PRISM Clinical Research, LLC., St. Paul, MN, USA (Study Number: BCT-100-000) and retrospectively registered on 7 December 2020 at ClinicalTrials.gov as NCT04655352. Subjects were screened to determine whether they met the inclusion criteria within 28 days prior to commencement of the 28 day test period. Salus Independent Review Board reviewed and approved the study protocol, the subject informed consent document, and daily GI symptom assessment questionnaire (PRISM Study 1516 Kinetics, Figure S2). All participants provided written informed consent to participate in this study prior to any study procedures being completed. This study was performed in accordance with the current version of the declaration of Helsinki (52nd WMA General Assembly, Edinburgh, Scotland, October 2000) and the International Conference on Harmonisation (ICH) guidelines on Good Clinical Practice (GCP).

On the first day of each week, all participants (*n* = 30) visited the clinical research unit for vital signs assessment and receipt of weekly test product (Days 1, 8, 15, and 22). Participants were administered a single dose of placebo (maltodextrin with excipients) orally BID (Day 1 through Day 7). All participants received a single dose of MB40 (250 mg capsule with maltodextrin and excipients and equal to 5 × 10^9^ CFU per capsule) administered orally BID with 240 mL of water for 21 days (Day 8 through Day 28). Participants took the first dose of each treatment week (Days 1, 8, 15 and 22) at the clinical research unit. The remaining doses were self-administered at home. Participants were instructed to take the dose at the same time each day and return any un-used test article.

Assessment of GI health was performed using self-reported daily GI questionnaires (Figure S2) and the Bristol Stool Chart (daily recorded for each bowel movement) starting on study Day 1 through study Day 28. Enrolled participants on study Day 1 received training on the GI questionnaires and Bristol Stool Chart diary. These reports were reviewed weekly during clinic visits.

Documentation of any surgical, or medical procedures and concomitant use of medications were collected throughout this study. Adverse events (AEs) were recorded throughout this study at each clinic visit. An AE is any untoward medical occurrence associated with the use of the test article whether or not considered test article related.

This study was monitored regularly (Day 8, 15, 22 and 29) for regulatory compliance, protocol adherence and completeness of data collection. The following data were collected and analyzed for safety and tolerability: adverse events (AEs), GI questionnaires and Bristol Stool Charts, physical examination findings, clinical laboratory tests, and vital signs assessments.

## 3. Results

### 3.1. Subsection Enterotoxin Screens (In Silico)

The potential of MB40 to produce toxins was evaluated in silico via nBLAST based on the presence of toxin-producing genes similar to those found in *B. cereus* and via BLASTx based on toxin proteins from *B. cereus*. The control genes and proteins, gatA and metG, yielded positive matches of 100% identity with 100% sequence coverage and 70% identity with 99% sequence coverage, respectively (Table S2). For the comparator toxin genes, multiple gene sequences from different species were used to strengthen the results. No significant similarities were found between the queried toxin sequences and the MB40 genome. Additionally, the results remain the same when utilizing BLASTx which compares translated DNA sequences to protein sequences (Table S3). The identified matches, namely NheA, B, C and entFM from *B. cereus*, were only partial matches across less than 20% of the toxin sequence (Table S2). These small fragment matches are not significant enough to conclude that MB40 contains the necessary gene clusters to produce these enterotoxins, which is confirmed by additional protein alignments (Table S3) [34].

To confirm the results of the nBLAST search, in silico PCR was completed using common primers for hbl, nhe, and bceT that have been verified in the literature [25,26]. The in silico PCR only yielded matches using the positive control 16S primers. No toxin genes were found in the MB40 genome during virtual PCR (Table S1). As before, the ability of this tool to identify the presence of the control sequences both in MB40 as well as in the *B. cereus* genome indicates a functional tool and thus the lack of toxin matches found in MB40 indicates an absence of such genes in the MB40 genome. The in silico PCR confirmed the gene nBLAST searches. Taken together, these data support the absence of harmful toxins genes in MB40.

### 3.2. Enterotoxin Screens (In Vitro)

In vitro enterotoxin screens were completed to assess whether MB40 cells produce potentially harmful toxins. First, two commercially available kits were used to test the spent growth media for the presence of two toxins commonly produced by *Bacillus* strains: *Bacillus* diarrheal enterotoxin (BDE) and *B. cereus* enterotoxin (BceT). Precedence for the use of these kits to test for the production of these two toxins by an organism via fermentation medium has been established [35]. Neither the BDE nor the BceT toxin were detected at their respective detection thresholds (1 ng/mL and 2 ng/mL) in the spent MB40 growth media. Under typical growth conditions, MB40 does not produce either toxin, which is corroborated by the absence of toxin genes in the MB40 genome.

### 3.3. Antibiotic Sensitivity Testing (AST)

Antibiotic sensitivity testing (AST) was completed per the standards established by the Clinical Laboratory Standards Institute [28] using antibiotics representing but not limited to antibiotic classes such as aminoglycosides, penicillins, glycopeptides, and macrolides. Based on diameter of inhibitions zones, MB40 was susceptible to 18 of the 21 antibiotics to which it was exposed (Table 1). MB40 was resistant to fosfomycin and was neither susceptible nor resistant to ampicillin and rifampin. 

As a follow up to this study, Minimum Inhibitory Concentration (MIC) values were measured for the eight antibiotics (vancomycin, gentamicin, kanamycin, streptomycin, erythromycin, clindamycin, tetracycline, and chloramphenicol) that the European Food Safety Authority (EFSA) determined to be of human and veterinary importance. EFSA does not require an ampicillin test when evaluating *Bacillus* strains [36]. MB40 was susceptible to all antibiotics except streptomycin (Table 2). The fact that MB40 is resistant to streptomycin is not a unique finding for *Bacillus* species. A study performed by Adimpong et.al. showed that out of 85 *Bacillus* species used for Sudanese bread production (*B. subtilis* subsp. subtilis (*n* = 29), *B. licheniformis* (*n* = 38) and *B. sonorensis* (*n* = 18)), all were resistant to streptomycin [37]. Additionally, the aminoglycoside nucleotidyltransferase which confers streptomycin resistance was stably present in the MB40 genome [Comprehensive Antibiotic Resistance Database data on file]. 

### 3.4. Oral Toxicity Study in Rats

Oral administration of MB40 to male and female Crl:CD(SD) rats at dosage levels of 500, 1000, and 2000 mg/kg/day equating to 1.71 − 2.18 × 10^10^, 3.38 − 4.33 × 10^10^, and 6.84 − 8.51 × 10^10^ CFU per day for 14 consecutive days resulted in no test article-related effects at any dosage level tested. No mortality and no test article-related effects were reported for any of the evaluated parameters at any dose of MB40. There were no test article-related clinical observations. Body weights (Tables S4 and S5) and food consumption (Tables S6 and S7) were unaffected by test article administration. 

Some statistically significant differences in hematology and coagulation parameters were reported when the control and test article-treated groups were compared but were considered non-test-article related because they were not dose dependent and were generally within the laboratory’s historical range (Table 3). Slightly higher mean prothrombin times were noted in all test article-treated male groups and the low-dose female group, but these increases did not occur in a dose-related manner and group means were generally within the laboratory’s historical control range of study means (14.1–18.1 seconds for males and 13.7–17.5 seconds for females). Higher mean alanine aminotransferase (ALT) values were noted in all test article-treated female groups (statistically significant at 500 and 2000 mg/kg bw/day) (Table 4) but there was no dose–response relationship and group means (26 to 29 U/L) were within the laboratory’s historical control range of study means (24–71 U/L). There was no effect of treatment on urinalysis of males or females (Table 5).

There were no test article-related macroscopic findings at the scheduled necropsy. All macroscopic findings noted were considered to be spontaneous and/or incidental in nature and unrelated to test article administration. Higher mean adrenal gland weights (absolute and/or relative to brain weight) were noted in the test article-treated male groups at 500 and/or 2000 mg/kg bw/day (Table 6) but were considered non-test article related because of the lack of a dose–response relationship and because group means were within the laboratory’s historical control range (absolute adrenal weight 0.0406–0.0795 g; relative to brain weight: 2.1072–3.9678 g). Additionally, higher mean testes weights (absolute and relative to brain weight) were noted in the 500 mg/kg/day group males. This difference also was not considered test article related due to the lack of a dose–response relationship and a mean value within the range of historical controls (absolute testes weight: 2.59–3.87 g; relative to brain weight: 132.998–194.337 g). 

Oral administration of MB40 to male and female Crl:CD(SD) rats at dosage levels of 500, 1000, and 2000 mg/kg bw/day (equivalent to 1.71 − 2.18 × 10^10^, 3.38 − 4.33 × 10^10^, and 6.84 − 8.51 × 10^10^ CFU per day) for 14 consecutive days was well tolerated at all dosage levels tested. The no-observed-adverse-effect level (NOAEL) for MB40 after was determined to be 2000 mg/kg bw/day (equivalent to 3.7 × 10^10^ CFU/kg bw/day), the highest dose tested. 

### 3.5. Human Clinical Safety and Tolerability Trial

**Figure 1:**
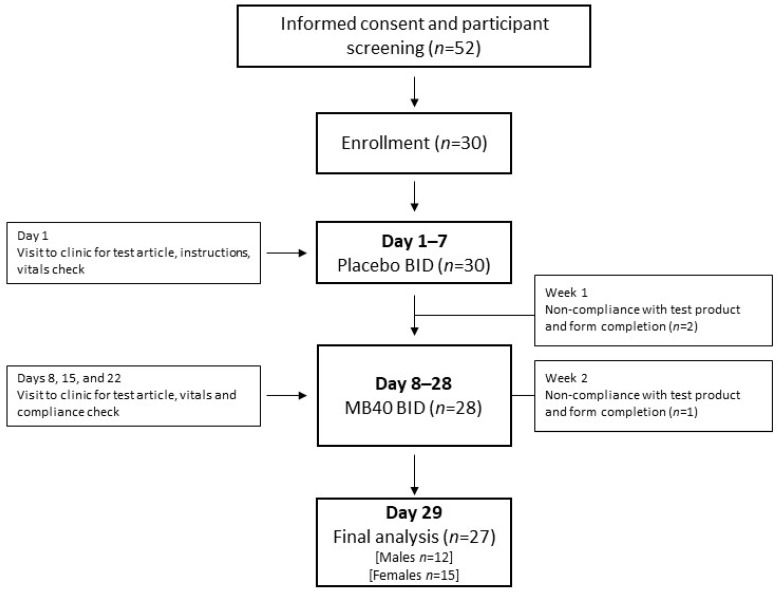
Human Safety and tolerability study design. BID = taken twice daily.

Fifty-two healthy participants were screened for this study and 30 participants were enrolled. Three total participants discontinued participation from this study after week 1 (two participants) and week 2 (one participant) due to non-compliance with test article and completion of the study forms (Figure 1). The completed participants consisted of 12 males and 15 females, with an average age of 35.3 ± 11.2 years and an average weight of 75.6 ± 15.4 kg. The overall test article compliance of the subjects that completed this study was 99.2% ± 3.3%.

There were no serious adverse events (AEs). There were five reported AEs during this study, all graded as level 1 (scale 1–4, with 4 being the most severe), and two were ascribed as likely related to the administration of the probiotic, neither of which required treatment (one instance of vomiting and one of chills both reported by the same subject on the same day during the middle of the treatment period and resolving within 31 h). There were no medically clinically significant changes based on physical examination findings, clinical laboratory results and vital signs.

There were no significant changes in the number of bowel movements per subject per week between the placebo week (average of 11.1 ± 4.6) and the three subsequent weeks when the test article was administered (week 2: 10.7 ± 3.6; week 3: 10.7 ± 3.8; week 4: 11.2 ± 4.3). Each participant’s Bristol stool form description was scored Type 1 (hard) through Type 7 (watery). The Bristol stool form score was consistent across all of the study weeks for each subject (average for the placebo week 1: 3.8 ± 0.1; averages for treatment weeks, week 2: 3.9 ± 0.1; week 3: 3.9 ± 0.1; week 4: 3.9 ± 0.2).

The symptoms reported on the daily GI questionnaires (e.g., abdominal bloating, abdominal pain, constipation, and flatulence) were few and of low severity (graded on a scale of 1—very mild to 10—extreme) such that statistical evaluation was not feasible. The symptoms during the treatment period generally occurred with similar or lower incidence and severity compared to the placebo week. None of the symptoms reported during the treatment period had an average severity over 6; typically, the average severity was less than 4 (Table 7). Additionally, while not statistically significant, an overall decrease in the average number of symptoms reported each week and the number of subjects reporting those symptoms was observed when comparing the treatment period to the placebo week.

Oral administration of MB40 at 10 × 10^9^ (10 billion) CFU/day for 21 days was well tolerated by healthy adults.

## 4. Discussion

Microbes play an important role in human health and wellness, as evidenced by a steady cadence of non-clinical and clinical scientific publications connecting healthy human physiology with our gut microbiome. With so many suggested health benefits tied to specific microorganisms, the popularity of probiotic supplementation has increased dramatically in the last decade. Strain safety is as important as the potential health benefits and both should be substantiated at the strain level. 

Pariza et.al. [23] proposed a path for evaluating the safety of probiotics in the form of a decision tree based on similar decision trees used worldwide. Laid out as a series of 13 questions, this decision tree focuses on safety at the individual strain level and dictates information that should be known and available for every probiotic strain. Pariza’s focus on making safety determinations based on strain-specific genomes rather than species-based assumptions is compelling. This decision tree includes strain characterization and genome sequencing, screening for undesirable attributes and metabolites, genetic modification, strain origin (e.g., occurrence in the food supply), and ongoing evaluation. Importantly, question 10 asks, “*Do scientific findings published since completion of the comprehensive peer-reviewed safety evaluation cited in question 9a continue to support the conclusion that the species, to which the strain belongs, is safe for use in food*?” This call to action for perpetual safety evaluation is paramount.

Since that 2015 call for iterative safety assessment, an Expert Panel of regulatory, industry, and research leaders advocated for increased quality and consistency in probiotic products, as well as improved consumer trust through transparent communications that include science-based assessments and third-party evaluations [39]. Curiously, though, this Expert Panel downplayed the importance of in vivo safety testing in animal models ahead of human studies perhaps due to the long history of safe food use and safe supplementation with *Lactobacillus* and *Bifidobacterium* spp. However, for candidate probiotic strains from spore-forming *Bacillus* spp., we suggest that at least a 14 day acute rat toxicity study is performed to evaluate a novel strain, and that at least a 90 day sub-chronic rat toxicity study is performed to evaluate a novel strain from a *Bacillus* sp. not yet approved as a novel dietary ingredient or for use in foods, before proceeding to a human safety and tolerability study. Exceptions to our guidance may apply as manufactured spore-forming *Bacillus* ssp. establish a history of safe supplementation similar to *Lactobacillus*. Nonetheless, widespread adoption of specific criteria for microorganisms to be considered a probiotic is also important. In 2020, global industry leaders presented four straightforward criteria for probiotics: (1) sufficiently characterized; (2) safe for the intended use; (3) supported by at least one positive human clinical trial; and (4) alive in the product at an efficacious dose throughout shelf life [40]. These criteria again highlight the need for strain-specific characterization of safety.

An additional benefit of the Pariza decision tree is that it addresses strain origin and provides guidance for strains isolated directly from a food source as well as those isolated from non-food sources such as the soil. In the case of a strain derived from a non-food source, question 13 says that “*Experimental evidence of safety is required. Such evidence may include, but is not necessarily limited to, studies in appropriate animal models, and clinical trials in humans*.” Experimental evidence of safety is needed but because the exact experimental evidence is not defined, scientists can work within current regulations to determine whether animal models and/or clinical studies are appropriate.

Aligning with the first two criteria—sufficiently characterized and safe for the intended use—in the simple decision tree presented by industry experts [40] and following Pariza’s extensive decision tree, the MB40 genome was sequenced and analyzed for undesirable attributes such as enterotoxin production and antibiotic resistance. Additionally, resources such as the Pathosystems Resource Integration Center (PATRIC) [41] were utilized to more fully characterize any virulence factors or mobile elements and annotate the MB40 genome (unpublished data). Similar genome mining was utilized for two *Lactobacillus* and two *Bifidobacterium* strains [42]. Despite the long history of safe use of species of *Lactobacillus* and *Bifidobacterium*, the authors presented on the importance of going beyond species characteristics to strain-specific safety requirements such as the absence of transferable antibiotic resistance which lends further credence to the approach taken with MB40. Additionally, because BLAST alignments and annotation databases can be limited predictive tools, in vitro screens also confirmed the absence of undesirable attributes such as *Bacillus* toxin production.

A short-term toxicology study in a rodent model was also completed prior to assessing tolerability in adult human subjects. During this rodent study, there were no MB40-related differences between the control and test groups in any measured parameters including hematological parameters, clinical chemistry, and organ weights. While some test groups had individual measurements such as prothrombin times that were statistically significant from the control in this study, all values fall within the laboratory’s historical control range of study means and were thus deemed not related to the test article. Based on the no-observed-adverse-effect level (NOAEL) of 2000 mg/kg bw/day (equivalent to 3.7 × 10^11^ CFU/kg bw/day or 6.84 − 8.51 × 10^10^ CFU/day in male and female rats), determined in the short-term repeated-dose rodent toxicity study with MB40, and a conservative 100-fold safety factor for inter- and intra-species differences, the acceptable daily intake level of *B. subtilis* MB40 for humans was calculated at 3.7 × 10^9^ CFU/kg bw/day (or 2.6 × 10^11^ (260 billion) CFU/day for a 70 kg person). Short-term oral toxicity studies in animals with other strains of *B*. *subtilis* [43,44], in which no adverse events were reported after repeated exposures to *B*. *subtilis*, are corroborated by the results of our toxicity study with *B. subtilis* MB40 and support the safety and appropriateness of the above calculated acceptable daily intake.

The ultimate safety assessment for the MB40 strain was the tolerability study in human subjects. Combined results from self-reported, daily GI questionnaires show no gross differences between the baseline placebo week and the subsequent 3 weeks of MB40 supplementation. If anything, there is a downward trend in gas and bloating with time, but the low number of instances of GI symptoms each week precluded statistical evaluation. The tolerated level of MB40 was determined to be 10 × 10^9^ CFU/day. This level was determined from consistent frequency of bowel movements throughout this study and the absence of medically significant changes based on physical examination, vital signs, and clinical laboratory results from study enrollment to completion. Demonstrated safety of MB40 supplementation in this study corroborates the results of published clinical safety studies with other strains of *B. subtilis* in which no adverse events were reported after repeated administration to human volunteers up to 10 × 10^9^ CFU/day [45,46].

## 5. Conclusions

The safety and tolerability of *Bacillus subtilis* MB40 were determined using the procedure outlined by Pariza and colleagues [23]. Based on the outcome of that decision tree approach for determining the safety of microbial cultures for consumption by humans and animals, including strain characterization and genome sequencing, screening for undesirable attributes and metabolites, and experimental evidence of safety by appropriately designed safety evaluation studies, *B. subtilis* MB40 does not pose human safety concerns at the acceptable daily intake of 3.7 × 10^9^ CFU/kg bw/day (i.e., 2.6 × 10^11^ (260 billion) CFU/day for a 70 kg adult).

## Figures and Tables

**Table 1:** Antibiotic Sensitivity Testing summary results for *B. subtilis* MB40.

Test Group	Disc Code	Inhibition Zone (mm)	Zone Interpretation
Aminoglycosides			
Kanamycin	K 30	35	S
Gentamicin	GM 10	33	S
Neomycin	N 30	29	S
Streptomycin	S 10	15	S
b-Lactams: Penicillins			
Penicillin	P 10	29	S
Ampicillin	AM 10	28	Int
Amoxicillin/Clavulanic Acid	AmC 30	31	S
b-Lactams: Cephems			
Cephalothin	CF 30	52	S
Cefotaxime	CTX 30	25	S
Cefaclor	CEC 30	50	S
Ceftriaxone	CRO 30	26	S
Fluorquinolones			
Ciprofloxacin	CIP 5	30	S
Fosfomycins			
Fosfomycin + Glucose-6-Phosphate	FOS 200	6	R
Folate Pathway Inhibitors			
Sulfamethoxazole Trimethorprim	SXT	32	S
Glycopeptides			
Vancomycin	Va 5	17	S
Macrolides, Lincosamides, Streptogramins			
Clindamycin	CC 2	22	S
Erythromycin	E 15	33	S
Quinupristin/Dalfopristin	SYN 15	20	S
Phenicols			
Chloramphenicol	C 30	30	S
Rifampin			
Rifampin	RA 5	18	Int
Tetracyclines			
Tetracycline	Te 30	19	S

S—susceptible; R—resistant; Int—intermediate.

**Table 2:** Minimum Inhibitory Concentration of Antibiotics for *B. subtilis* MB40.

Antibiotic	MIC (µg/mL)			
	EFSA Cut-off Values for *Bacillus* [36]	MB40	*S. aureus*	*E. faecalis*
Vancomycin	4	0.5	1	
Gentamicin	4	0.125	0.25	
Kanamycin	8	1	2	
Streptomycin	8	>32	N/A	32 *
Erythromycin	4	0.125	0.25	
Clindamycin	4	2	0.125	
Tetracycline	8	4	0.5	
Chloramphenicol	8	4	8	

* Inhibition at 32 µg/mL can be extrapolated to indicate susceptibility of streptomycin at 1000 µg/mL and a lack of high-level aminoglycoside resistance per the CLSI [38].

**Table 3:** Effect of 14 day oral administration of *B. subtilis* MB40 on hematological parameters in male and female rats.

Parameter	Units	Group and Dose (mg/kg bw/day)							
		Males				Females			
		Control *n ^2^* = 9	500 *n* = 10	1000 *n ^2^* = 9	2000 *n* = 10	Control *n* = 10	500 *n ^2^* = 8	1000 *n ^2^* = 8	2000 *n* = 10
WBC	×10^3^/µL	10.36 ± 1.70	10.22 ± 2.47	10.27 ± 2.43	9.91 ± 2.24	6.84 ± 2.08	6.86 ± 1.79	7.10 ± 1.77	7.66 ± 2.00
RBC	×10^6^/µL	7.45 ± 0.35	7.83 ± 0.29 *	7.71 ± 0.34	7.53 ± 0.28	7.98 ± 0.33	7.73 ± 0.52	7.89 ± 0.37	7.86 ± 0.36
HGB	g/dL	15.2 ± 0.56	15.6 ± 0.62	15.5 ± 0.57	15.2 ± 0.56	16.1 ± 0.74	15.6 ± 0.49	15.8 ± 0.41	15.7 ± 0.59
HCT	%	46.2 ± 1.65	47.7 ± 2.04	47.3 ± 1.42	46.5 ± 1.60	47.2 ± 2.68	45.7 ± 1.69	46.7 ± 1.12	46.8 ± 1.92
MCV	fL	62.2 ± 1.23	60.9 ± 2.25	61.4 ± 1.19	61.8 ± 1.58	59.1 ± 1.29	59.3 ± 2.23	59.3 ± 1.66	59.6 ± 1.68
MCH	pg	20.4 ± 0.45	19.9 ± 0.81	20.2 ± 0.47	20.3 ± 0.55	20.1 ± 0.39	20.3 ± 0.96	20.1 ± 0.66	20.0 ± 0.51
MCHC	g/dL	32.9 ± 0.52	32.7 ± 0.61	32.8 ± 0.65	32.8 ± 0.51	34.1 ± 0.54	34.2 ± 0.64	33.9 ± 0.57	33.6 ± 0.44
RDW	%	12.4 ± 0.30	12.6 ± 0.41	12.6 ± 0.57	12.4 ± 0.42	11.3 ± 0.35	11.3 ± 0.35	11.2 ± 0.23	11.3 ± 0.32
HDW	g/dL	1.96 ± 0.07	1.96 ± 0.25	1.86 ± 0.13	1.90 ± 0.07	1.97 ± 0.10	1.92 ± 0.12	1.95 ± 0.11	1.93 ± 0.08
MPV	fL	7.44 ± 0.37	7.39 ± 0.23	7.39 ± 0.28	7.22 ± 0.38	6.95 ± 0.31	6.83 ± 0.23	6.98 ± 0.36	6.87 ± 0.26
PLT	×10³/µL	948 ± 108.6	929 ± 171.3	762 ± 240.1	997 ± 212.9	898 ± 155.2	1007 ± 139.5	887 ± 310.2	1047 ± 119.5
ANEU	×10³/µL	1.38 ± 0.22	2.09 ± 1.99	1.79 ± 0.68	1.51 ± 0.22	1.04 ± 0.26	1.26 ± 0.41	1.18 ± 0.40	1.21 ± 0.43
ALYM	×10³/µL	8.59 ± 1.48	7.68 ± 1.85	8.09 ± 1.90	8.02 ± 2.17	5.55 ± 2.17	5.37 ± 1.67	5.68 ± 1.60	6.13 ± 1.88
AMON	×10³/µL	0.23 ± 0.07	0.23 ± 0.10	0.21 ± 0.07	0.22 ± 0.09	0.10 ± 0.02	0.11 ± 0.04	0.10 ± 0.03	0.16 ± 0.10
AEOS	×10³/µL	0.07 ± 0.03	0.13 ± 0.07 *	0.11 ± 0.07	0.07 ± 0.03	0.11 ± 0.06	0.07 ± 0.02	0.09 ± 0.05	0.09 ± 0.06
ABAS	×10³/µL	0.02 ± 0.01	0.02 ± 0.01	0.02 ± 0.01	0.02 ± 0.01	0.01 ± 0.01	0.01 ± 0.01	0.01 ± 0.01	0.02 ± 0.01
ALUC	×10³/µL	0.07 ± 0.03	0.06 ± 0.02	0.06 ± 0.01	0.07 ± 0.03	0.04 ± 0.02	0.04 ± 0.01	0.04 ± 0.02	0.06 ± 0.03
ARET	×10³/µL	257 ± 25.8	290 ± 83.2	252 ± 37.3	242 ± 35.7	185 ± 38.4	195 ± 33.9	177 ± 20.2	188.8 ± 30.9
PT ^1^	sec	16.5 ± 0.54	17.8 ± 0.71 ^†^	18.2 ± 0.61 ^†^	18.0 ± 0.78 ^†^	16.0 ± 0.81	16.9 ± 1.09 *	16.5 ± 0.66	16.9 ± 0.63
APTT ^1^	sec	11.7 ± 0.80	10.9 ± 0.87	10.9 ± 0.79	11.0 ± 1.07	11.5 ± 1.12	11.2 ± 0.58	11.6 ± 1.17	12.1 ± 1.51

Values are the mean ± standard deviation. ^1^ *n* = 10. ^2^ *n* < 10 because blood samples clotted before analysis. * Significantly different from the control group at *p* < 0.05 using Dunnett’s test. ^†^ Significantly different from the control group at *p* < 0.01 using Dunnett’s test. ABAS = absolute basophil; AEOS = absolute eosinophil; ALUC = absolute large unstained cell; ALYM = absolute lymphocyte; AMON = absolute monocyte; ANEU = absolute neutrophil (all forms); APTT = activated partial thromboplastin time; ARET = absolute reticulocyte; fL = femtoliters; g/dL = grams/deciliter; HCT = hematocrit; HDW = hemoglobin distribution width; HGB = hemoglobin; MCH = mean corpuscular hemoglobin; MCHC = mean corpuscular hemoglobin concentration; MCV = mean corpuscular volume; MPV = mean platelet volume; PLT = platelet count; PT = prothrombin time; RBC = red blood cell count; RDW = red cell distribution width; WBC = white blood cell count.

**Table 4:** Effect of 14 day oral administration of *B. subtilis* MB40 on clinical chemistry parameters in male and female rats.

Parameter	Units	Group and Dose (mg/kg bw/day)							
		Males				Females			
		Control *n* = 10	500 *n* = 10	1000 *n* = 10	2000 *n* = 10	Control *n* = 10	500 *n* = 10	1000 *n* = 10	2000 *n* = 10
ALB	g/dL	3.7 ± 0.13	3.8 ± 0.16	3.6 ± 0.12	3.6 ± 0.13	4.1 ± 0.18	4.1 ± 0.13	4.2 ± 0.13	4.2 ± 0.16
TP	g/dL	6.0 ± 0.23	6.0 ± 0.24	5.9 ± 0.19	5.8 ± 0.26	6.5 ± 0.26	6.5 ± 0.21	6.6 ± 0.21	6.5 ± 0.23
GLOB	g/dL	2.3 ± 0.14	2.3 ± 0.13	2.3 ± 0.16	2.2 ± 0.13	2.4 ± 0.11	2.4 ± 0.09	2.4 ± 0.13	2.4 ± 0.16
ALB/GLOB		1.66 ± 0.10	1.67 ± 0.10	1.63 ± 0.13	1.62 ± 0.06	1.72 ± 0.08	1.73 ± 0.07	1.77 ± 0.11	1.76 ± 0.14
ALT	U/L	43 ± 10.0	39 ± 3.9	38 ± 3.4	39 ± 4.2	24 ± 3.0	29 ± 4.4 *	26 ± 4.1	29 ± 3.6 *
AST	U/L	115 ± 16.7	109 ± 34.7	106 ± 21.7	102 ± 20.7	104 ± 18.4	125 ± 30.7	103 ± 25.0	116 ± 21.6
ALP	U/L	256 ± 48.5	291 ± 53.1	291 ± 60.3	260 ± 45.3	151 ± 35.7	167 ± 53.7	153 ± 30.6	151 ± 52.5
GGT	U/L	0.00 ± 0.00	0.00 ± 0.00	0.00 ± 0.00	0.00 ± 0.00	0.00 ± 0.00	0.00 ± 0.00	0.00 ± 0.00	0.00 ± 0.00
SDH	U/L	10 ± 5.6	9 ± 2.7	8 ± 2.5	7 ± 2.2	12 ± 7.8	9 ± 4.6	10 ± 3.2	12 ± 7.4
TBIL	mg/dL	0.00 ± 0.00	0.01 ± 0.03	0.00 ± 0.00	0.00 ± 0.00	0.00 ± 0.00	0.00 ± 0.00	0.00 ± 0.00	0.00 ± 0.00
CREAT	mg/dL	0.22 ± 0.03	0.22 ± 0.03	0.21 ± 0.03	0.20 ± 0.03	0.33 ± 0.07	0.32 ± 0.04	0.30 ± 0.02	0.34 ± 0.06
BUN	mg/dL	12.5 ± 0.83	12.6 ± 1.55	12.4 ± 1.66	12.6 ± 1.49	15.8 ± 1.69	17.4 ± 1.70	16.9 ± 3.12	16.8 ± 1.87
Ca	mg/dL	10.7 ± 0.28	10.6 ± 0.33	10.5 ± 0.26	10.5 ± 0.27	10.3 ± 0.16	10.2 ± 0.16	10.3 ± 0.21	10.3 ± 0.25
Cl	mEq/L	104 ± 1.1	104 ± 1.4	104 ± 1.2	104 ± 0.7	108 ± 1.0	108 ± 0.8	107 ± 1.5	106 ± 2.0
PHOS	mg/dL	9.9 ± 0.42	9.7 ± 0.53	9.7 ± 0.57	9.4 ± 0.31	7.5 ± 0.64	7.7 ± 0.57	7.4 ± 0.66	8.0 ± 0.84
K	mEq/L	5.63 ± 0.46	5.40 ± 0.43	5.37 ± 0.42	5.38 ± 0.28	5.31 ± 0.56	5.08 ± 0.56	5.04 ± 0.34	5.11 ± 0.40
Na	mEq/L	144 ± 0.7	144 ± 1.2	144 ± 1.2	144 ± 1.2	145 ± 1.4	145 ± 0.7	144 ± 1.1	144 ± 1.2
GLU	mg/dL	97 ± 4.8	106 ± 13.3	101 ± 8.3	104 ± 11.0	105 ± 6.9	106 ± 8.3	106 ± 7.9	103 ± 12.7
CHOL	mg/dL	67 ± 12.7	72 ± 13.6	64 ± 8.1	64 ± 12.1	62 ± 15.7	55 ± 7.4	60 ± 16.3	64 ± 9.4
TRIG	mg/dL	60 ± 17.0	62 ± 19.0	62 ± 21.5	56 ± 10.9	25 ± 3.5	22 ± 4.6	26 ± 7.1	26 ± 8.1

Values are the mean ± standard deviation. * Significantly different from the control group at *p* < 0.05 using Dunnett’s test. ALB = albumin; ALP = alkaline phosphatase; ALT = alanine aminotransferase; AST = aspartate aminotransferase; BUN = urea nitrogen; Ca = calcium; CHOL = cholesterol; Cl = chloride; CREAT = creatinine; GGT = gamma glutamyl transferase; GLOB = globulin; GLU = glucose; K = potassium; mg/dL = milligrams/deciliter; mEq = milliequivalents; Na = sodium; PHOS = inorganic phosphorous; SDH = sorbitol dehydrogenase; TBIL = total bilirubin; TP = total protein; TRIG = triglycerides; U/L = international unit/Liter.

**Table 5:** Effect of 14 day oral administration of *B. subtilis* MB40 on urinalysis parameters in male and female rats.

Parameter	Units	Group and Dose (mg/kg bw/day)							
		Males				Females			
		Control *n* = 10	500 *n* = 10	1000 *n* = 10	2000 *n* = 10	Control *n* = 10	500 *n* = 10	1000 *n* = 10	2000 *n* = 10
SPGRAV		1.023 ± 0.011	1.028 ± 0.013	1.028 ± 0.012	1.032 ± 0.016	1.032 ± 0.014	1.034 ± 0.016	1.029 ± 0.007	1.027 ± 0.009
pH		7.2 ± 0.48	7.3 ± 0.54	7.3 ± 0.42	7.1 ± 0.37	6.0 ± 0.28	5.9 ± 0.30	6.0 ± 0.24	5.9 ± 0.22
UROBIL	mg/dL	0.2 ± 0.00	0.2 ± 0.00	0.2 ± 0.00	0.2 ± 0.00	0.2 ± 0.00	0.2 ± 0.00	0.2 ± 0.00	0.2 ± 0.00
TVOL	mL	9.1 ± 3.70	9.1 ± 5.93	8.2 ± 4.85	6.8 ± 3.91	6.1 ± 4.86	7.1 ± 7.39	6.0 ± 2.49	7.0 ± 4.18

Values are the mean ± standard deviation. SPGRAV = specific gravity; TVOL = total volume; UROBIL = urobilinogen.

**Table 6:** Effect of 14 day oral administration of *B. subtilis* MB40 on organ weights of male and female rats.

Parameter	Units	Group and Dose (mg/kg bw/day)							
		Males				Females			
		Control *n* = 10	500 *n* = 10	1000 *n* = 10	2000 *n* = 10	Control *n* = 10	500 *n* = 10	1000 *n* = 10	2000 *n* = 10
TBW	g	311 ± 28.8	322 ± 24.2	318 ± 24.0	312 ± 20.2	204 ± 15.2	206 ± 15.4	208 ± 11.4	208 ± 17.5
Adrenals	g	0.048 ± 0.006	0.056 ± 0.005 ^†^	0.054 ± 0.004	0.055 ± 0.007 *	0.061 ± 0.008	0.066 ± 0.007	0.060 ± 0.007	0.066 ± 0.007
Adrenals/TBW	g/100 g	0.016 ± 0.002	0.018 ± 0.002	0.017 ± 0.001	0.018 ± 0.002	0.030 ± 0.003	0.032 ± 0.004	0.029 ± 0.004	0.032 ± 0.002
Adrenals/TBRW	g/100 g	2.550 ± 0.282	2.965 ± 0.310 ^†^	2.769 ± 0.201	2.836 ± 0.344	3.306 ± 0.434	3.545 ± 0.384	3.232 ± 0.336	3.620 ± 0.314
Brain	g	1.88 ± 0.08	1.91 ± 0.07	1.94 ± 0.08	1.94 ± 0.06	1.85 ± 0.07	1.87 ± 0.08	1.86 ± 0.07	1.83 ± 0.07
Brain/TBW	g/100 g	0.609 ± 0.052	0.595 ± 0.050	0.614 ± 0.048	0.622 ± 0.038	0.910 ± 0.053	0.910 ± 0.081	0.897 ± 0.044	0.885 ± 0.066
Heart	g	1.32 ± 0.15	1.39 ± 0.17	1.32 ± 0.16	1.31 ± 0.10	0.90 ± 0.10	0.91 ± 0.06	0.99 ± 0.14	0.94 ± 0.09
Heart/TBW	g/100 g	0.424 ± 0.034	0.431 ± 0.037	0.414 ± 0.038	0.420 ± 0.026	0.442 ± 0.034	0.441 ± 0.030	0.478 ± 0.083	0.451 ± 0.040
Heart/TBRW	g/100 g	69.964 ± 8.010	72.815 ± 8.187	67.761 ± 7.615	67.699 ± 5.146	48.757 ± 4.738	48.616 ± 3.470	53.345 ± 8.818	51.131 ± 4.620
Kidneys	g	2.71 ± 0.27	2.74 ± 0.22	2.69 ± 0.18	2.76 ± 0.27	1.69 ± 0.13	1.78 ± 0.19	1.73 ± 0.08	1.77 ± 0.14
Kidneys/TBW	g/100 g	0.871 ± 0.051	0.852 ± 0.039	0.848 ± 0.054	0.882 ± 0.049	0.829 ± 0.042	0.863 ± 0.075	0.836 ± 0.031	0.851 ± 0.065
Kidneys/TBRW	g/100 g	143.784 ± 13.822	144.088 ± 14.090	138.610 ± 10.194	142.450 ± 14.074	91.257 ± 4.822	95.578 ± 11.958	93.285 ± 4.083	96.257 ± 6.595
Liver	g	12.03 ± 1.44	12.17 ± 1.06	11.69 ± 1.12	11.83 ± 1.24	7.30 ± 0.75	7.70 ± 0.63	7.59 ± 0.61	7.91 ± 0.66
Liver/TBW	g/100 g	3.862 ± 0.224	3.788 ± 0.304	3.677 ± 0.167	3.784 ± 0.253	3.569 ± 0.216	3.738 ± 0.216	3.655 ± 0.208	3.810 ± 0.255
Liver/TBRW	g/100 g	638.137 ± 65.444	639.713 ± 64.238	602.730 ± 59.382	611.639 ± 69.043	393.042 ± 28.612	413.189 ± 36.288	407.813 ± 22.574	431.661 ± 31.625
Pituitary	g	0.012 ± 0.002	0.014 ± 0.002	0.014 ± 0.002	0.0142 ± 0.003	0.015 ± 0.002	0.015 ± 0.002	0.015 ± 0.002	0.016 ± 0.002
Pituitary/TBW	g/100 g	0.004 ± 0.001	0.004 ± 0.000	0.004 ± 0.001	0.005 ± 0.001 *	0.007 ± 0.001	0.007 ± 0.002	0.007 ± 0.001	0.008 ± 0.001
Pituitary/TBRW	g/100 g	0.621 ± 0.094	0.720 ± 0.104	0.696 ± 0.106	0.730 ± 0.126	0.806 ± 0.107	0.812 ± 0.143	0.813 ± 0.122	0.875 ± 0.087
Spleen	g	0.73 ± 0.13	0.78 ± 0.18	0.76 ± 0.12	0.70 ± 0.12	0.48 ± 0.12	0.47 ± 0.08	0.50 ± 0.09	0.46 ± 0.07
Spleen/TBW	g/100 g	0.233 ± 0.035	0.241 ± 0.048	0.237 ± 0.026	0.223 ± 0.032	0.234 ± 0.046	0.229 ± 0.035	0.242 ± 0.043	0.221 ± 0.030
Spleen/TBRW	g/100 g	38.648 ± 7.350	40.779 ± 9.099	38.966 ± 6.884	36.031 ± 5.488	25.898 ± 5.939	25.353 ± 4.689	26.979 ± 4.793	25.044 ± 3.166
Thymus	g	0.672 ± 0.159	0.725 ± 0.156	0.711 ± 0.164	0.668 ± 0.119	0.542 ± 0.112	0.573 ± 0.115	0.526 ± 0.097	0.489 ± 0.084
Thymus/TBW	g/100 g	0.215 ± 0.043	0.225 ± 0.042	0.223 ± 0.044	0.213 ± 0.028	0.264 ± 0.046	0.278 ± 0.052	0.253 ± 0.041	0.236 ± 0.047
Thymus/TBRW	g/100 g	35.728 ± 8.575	37.924 ± 7.416	36.619 ± 8.324	34.506 ± 6.080	29.092 ± 5.263	30.818 ± 6.590	28.233 ± 4.811	26.699 ± 4.758
Thryoid + PTH	g	0.015 ± 0.002	0.017 ± 0.003	0.017 ± 0.003	0.016 ± 0.003	0.015 ± 0.002	0.015 ± 0.002	0.013 ± 0.002	0.014 ± 0.002
Thryoid + PTH/TBW	g/100 g	0.005 ± 0.001	0.006 ± 0.001	0.005 ± 0.001	0.005 ± 0.001	0.008 ± 0.001	0.007 ± 0.001	0.006 ± 0.001 *	0.007 ± 0.001
Thryoid + PTH/TBRW	g/100 g	0.805 ± 0.110	0.908 ± 0.157	0.887 ± 0.155	0.812 ± 0.136	0.822 ± 0.084	0.788 ± 0.112	0.722 ± 0.126	0.758 ± 0.090
Epididymides	g	0.59 ± 0.06	0.62 ± 0.06	0.65 ± 0.04	0.62 ± 0.07				
Epididymides/TBW	g/100 g	0.190 ± 0.019	0.194 ± 0.021	0.205 ± 0.023	0.201 ± 0.032				
Epididymides/TBRW	g/100 g	31.294 ± 2.245	32.786 ± 3.976	33.387 ± 2.866	32.301 ± 4.294				
Sem Ves+Prostate	g	1.76 ± 0.20	1.75 ± 0.21	1.88 ± 0.15	1.84 ± 0.22				
Sem Ves+ Prostate/TBW	g/100 g	0.569 ± 0.083	0.543 ± 0.050	0.595 ± 0.071	0.593 ± 0.086				
Sem Ves+ Prostate/TBRW	g/100 g	93.280 ± 10.280	91.923 ± 11.384	96.746 ± 6.438	95.193 ± 10.821				
Testes (g)	g	2.84 ± 0.26	3.13 ± 0.27 *	3.05 ± 0.18	2.92 ± 0.21				
Testes/TBW	g/100 g	0.915 ± 0.076	0.977 ± 0.112	0.963 ± 0.070	0.937 ± 0.065				
Testes/TBRW	g/100 g	150.543 ± 10.304	164.380 ± 13.689 *	157.245 ± 11.300	151.047 ± 11.821				
Ovaries	g					0.116 ± 0.019	0.127 ± 0.016	0.119 ± 0.014	0.123 ± 0.024
Ovaries/TBW	g/100 g					0.057 ± 0.007	0.062 ± 0.008	0.057 ± 0.007	0.059 ± 0.009
Ovaries/TBRW	g/100 g					6.274 ± 1.014	6.852 ± 1.064	6.398 ± 0.696	6.706 ± 1.153
Uterus	g					0.61 ± 0.16	0.49 ± 0.06	0.53 ± 0.22	0.54 ± 0.16
Uterus/TBW	g/100 g					0.299 ± 0.082	0.239 ± 0.038	0.254 ± 0.100	0.263 ± 0.089
Uterus/TBRW	g/100 g					32.838 ± 8.568	26.256 ± 3.783	28.250 ± 10.787	29.496 ± 8.388

Values are the mean ± standard deviation. Relative organ weights (organ/100 g TBW or 100 g TBRW) presented in the table are times 1000. PTH = parathyroid; Sem Ves = seminal vesicles; TBW = terminal body weight; TBRW = terminal brain weight. * Significantly different from the control group at *p* < 0.05 using Dunnett’s test. ^†^ Significantly different from the control group at *p* < 0.01 using Dunnett’s test.

**Table 7:** Summary of GI symptom severity ratings (mean and S.D.) in healthy adults given *B. subtilis* MB40.

Study Week	Severity Score	Nausea	Vomiting	Heartburn	Abdominal Bloating	Indigestion	Upper Abdominal Pain	Lower Abdominal Pain	Diarrhea	Constipation	Flatulence
1 (Placebo)	Mean *(S.D.)*	6.0 *(NA)* *n* = 1	8.0 *(NA)* *n* = 1	2.0 *(1.4)* *n* = 2	3.9 *(1.8)* *n* = 11	1.0 *(NA)* *n* = 1	3.0 *(NA)* *n* = 1	3.5 *(1.3)* *n* = 4	NA *(NA)* *n* = 0	3.8 *(1.8)* *n* = 14	2.7 *(1.3)* *n* = 36
2	Mean *(S.D.)*	NA *(NA)* *n* = 0	NA *(NA)* *n* = 0	1.0 *(0.0)* *n* = 2	3.4 *(2.2)* *n* = 9	NA *(NA)* *n* = 0	NA *(NA)* *n* = 0	2.4 *(0.9)* *n* = 5	6.0 *(NA)* *n* = 1	3.2 *(1.2)* *n* = 19	3.1 *(1.7)* *n* = 20
3	Mean *(S.D.)*	1.0 *(0.0)* *n* = 3	NA *(NA)* *n* = 0	NA *(NA)* *n* = 0	2.0 *(1.0)* *n* = 7	3.0 *(NA)* *n* = 1	NA *(NA)* *n* = 0	NA *(NA)* *n* = 0	NA *(NA)* *n* = 0	2.2 *(1.1)* *n* = 19	2.4 *(1.1)* *n* = 17
4	Mean *(S.D.)*	NA *(NA)* *n* = 0	NA *(NA)* *n* = 0	NA *(NA)* *n* = 0	2.3 *(0.4)* *n* = 7	NA *(NA)* *n* = 0	NA *(NA)* *n* = 0	3.3 *(0.6)* *n* = 3	1.0 *(NA)* *n* = 1	2.2 *(0.8)* *n* = 13	2.8 *(1.5)* *n* = 13

NA = not applicable; (*NA*) = Standard deviation cannot be calculated *n* ≤ 1 subject reported symptom.

## Data Availability

Data not presented within the article or Supplementary Materials is available upon request from the corresponding author. The data are not publicly available due to privacy.

## Supplementary Materials

The following are available online at https://www.mdpi.com/2072-6643/13/3/733/s1, Figure S1: Short-term tox design schematic; Figure S2: GI symptom questionnaire; Table S1: PCR primers and results summary; Table S2: nBLAST results; Table S3: BLASTx results; Table S4: Body weights, males; Table S5: Body weights, females; Table S6: Food consumption, males; Table S7: Food consumption, females.
