# Supplementary material for: Safety Assessment of Bacillus subtilis MB40 for Use in Foods and Dietary Supplements
Source: Nutrients. 2021 Feb 25;13(3):733. doi: 10.3390/nu13030733 (PMC7996492; doi:10.3390/nu13030733)
Supplement: Supplementary file 1 [file nutrients-13-00733-s001.zip › MB40 Safety and Tolerability Figure S1 Tox design-210130.docx]

Figure S1: Short-term Spague Dawley Rat Oral Toxicity Study Design

| Acclimation (12-day) | |
| --- | --- |
|  |  |
| Assignment of animals to study groups | |
|  |  |
| 10 animals/sex/group (Groups 1-4) treated for 14 consecutive days | |
|  |  |
| Detailed physical examinations performed weekly (± 2 days) during the study period and on the day of the scheduled necropsy;  Clinical observations recorded daily at the time of dose administration and 1-2 hours following dose administration;  Body weights recorded weekly (± 2 days) during the study period and on the day prior to and day of the scheduled necropsy;  Individual food weights recorded weekly (± 2 days) throughout the study period | |
|  |  |
| Clinical pathology evaluations conducted on the day of the scheduled necropsy (study day 14) for all animals | |
|  |  |
| Necropsies performed on all animals on study day14;  Selected organs weighed;  Selected tissues from all animals stored in the appropriate fixative for possible future histopathologic analysis | |
