# Supplementary material for: Safety Assessment of Bacillus subtilis MB40 for Use in Foods and Dietary Supplements
Source: Nutrients. 2021 Feb 25;13(3):733. doi: 10.3390/nu13030733 (PMC7996492; doi:10.3390/nu13030733)
Supplement: Supplementary file 1 [file nutrients-13-00733-s001.zip › MB40 Safety and Tolerability Figure S2 GI symptom questionnaire.docx]

**Daily GI Symptom Assessment Questionnaire**

The questions below are about side effects in your gastrointestinal system (nausea, abdominal pain, bloating, vomiting, gas diarrhea, constipation or indigestion) after taking the study product.

You may experience no GI side effects, have just one new symptom or worsening of a previous state, or have several side effects at the same time.

**DATE _____/_____/_____**

1. Did you experience nausea? **YES NO**

If YES, please rate on a scale of 1 (very mild) to 10 (extreme) ________

What time did the nausea start? _____________ When did it end? ____________

1. Did you experience vomiting? **YES NO**

If YES, please rate on a scale of 1 (very mild) to 10 (extreme) ________

What time did the vomiting start? _____________ When did it end? ____________

1. Did you experience heartburn? **YES NO**

If YES, please rate on a scale of 1 (very mild) to 10 (extreme) ________

What time did the heartburn start? _____________ When did it end? ____________

1. Did you experience abdominal bloating? **YES NO**

If YES, please rate on a scale of 1 (very mild) to 10 (extreme) ________

What time did the abdominal bloating start? _____________

When did it end? ____________

1. Did you experience indigestion? **YES NO**

If YES, please rate on a scale of 1 (very mild) to 10 (extreme) ________

What time did the indigestion start? _____________ When did it end? ____________

1. Did you experience upper abdominal pain? **YES NO**

If YES, please rate on a scale of 1 (very mild) to 10 (extreme) ________

What time did the upper abdominal pain start? _____________

When did it end? ____________

1. Did you experience lower abdominal pain? **YES NO**

If YES, please rate on a scale of 1 (very mild) to 10 (extreme) ________

What time did the lower abdominal pain start? _____________

When did it end? ____________

**GI Symptom Assessment Questionnaire**

**DATE _____/_____/_____**

1. Did you experience diarrhea? **YES NO**

If YES, please rate on a scale of 1 (very mild) to 10 (extreme) ________

What time did the diarrhea start? _____________ When did it end? ____________

1. Did you experience constipation (difficulty passing stool, hard stool, or no stool >48 hours)? **YES NO**

            If YES, please rate on a scale of 1 (very mild) to 10 (extreme) ________

            What time did the constipation start? _____________ When did it end? ____________

10. Did you experience flatulence (gas)? **YES NO**

If YES , please rate on a scale of 1 (very mild) to 10 (extreme) ________

What time did the flatulence start? _____________ When did it end? ____________

**Reviewed by Prism Staff Initials ________ Date _____/_____/_____**
