# Supplementary material for: Safety Assessment of Bacillus subtilis MB40 for Use in Foods and Dietary Supplements
Source: Nutrients. 2021 Feb 25;13(3):733. doi: 10.3390/nu13030733 (PMC7996492; doi:10.3390/nu13030733)
Supplement: Supplementary file 1 [file nutrients-13-00733-s001.zip › MB40 Safety and Tolerability Table S1 PCR.docx]

Table S1. In silico PCR primers and summary results for enterotoxin screening for B. subtilis MB40

| **Gene** | **Primer (5’ to 3’)*** | **MB40 Genome Position** | **Gene length** |
| --- | --- | --- | --- |
| Positive controls for 16S | 27F: AGAGTTTGATCMTGGCTCAG | Node 11, position 31 | 1512bp |
|  | 1492R: CGGTTACCTTGTTACGACTT |  |  |
|  | 785F: GGATTAGATACCCTGGTA | Node 11, position 815 | 728bp |
|  | 1492R: CGGTTACCTTGTTACGACTT |  |  |
| Positive control  spoIVA | F: GCAGAACGAACAGGCGG  R: GTTCTTTTAATTTATA | Node 18, position 217,709 | 1392 bp |
| hblA | F: AAGCAATGGAATACAATGGG | No bands amplified | N/A |
|  | R: AGAATCTAAATCATGCCACTGC |  |  |
| hblC | F: GATACCAATGTGGCAACTGC | No bands amplified | N/A |
|  | R: TTGAGACTGCTCG(T,C)TAGTTG |  |  |
| hblD | F: ACCGGTAACACTATTCATGC | No bands amplified | N/A |
|  | R: GAGTCCATATGGCTTAGATGC |  |  |
| nheA | F: TACGCTAAGGAGGGGCA | No bands amplified | N/A |
|  | R: GTTTTTATTGCTTCATCGGCT |  |  |
| nheB | F: CTATCAGCACTTATGGCAG | No bands amplified | N/A |
|  | R: ACTCCTAGCGGTGTTCC |  |  |
| nheC | F: CGGTAGTGATTGCTGGG | No bands amplified | N/A |
|  | R: CAGCATTCGTACTTGCCAA |  |  |
| bceT | F: CGTATCGGTCGTTCACTCGG | No bands amplified | N/A |
|  | R: TTTCTTTCCCGCTTGCCTTT |  |  |
| hblA 874bp | F: GCTAATGTAGTTTCACCTGTAGC | No bands amplified | N/A |
|  | R: AATCATGCCACTGCGTGGAC |  |  |
| bceT 428bp | F: TTACATTACCAGGACGTGCTT | No bands amplified | N/A |
|  | R: TGTTTGTGATTGTAATTCAGG |  |  |
| entFM 1269bp | F: ATGAAAAAAGTAATTTGCAGG | No bands amplified | N/A |
|  | R: TTAGTATGCTTTTGTGTAACC |  |  |

*F denotes forward primer and R denotes the reverse primer

N/A – not applicable
