# Supplementary material for: Safety Assessment of Bacillus subtilis MB40 for Use in Foods and Dietary Supplements
Source: Nutrients. 2021 Feb 25;13(3):733. doi: 10.3390/nu13030733 (PMC7996492; doi:10.3390/nu13030733)
Supplement: Supplementary file 1 [file nutrients-13-00733-s001.zip › MB40 Safety and Tolerability Table S2 nBLAST.docx]

Table S2. Summary of nBLAST screening results for *Bacillus* toxin genes in MB40

| **Gene** | **Organism** | **Gene ID** | **Max Score** | **% coverage** | **E-value** | **% Identical** |
| --- | --- | --- | --- | --- | --- | --- |
| gatA | *B. subtilis* | 938748 | 2630 | 100% | 0.00 | 100% |
| metG | *B. cereus* | 1202396 | 895 | 99% | 0.00 | 70% |
| HblA | *B. licheniformis* | KM514479.1 | No significant similarity found | | | |
| HblA | *B. cereus* | KF681259.1 | No significant similarity found | | | |
| HblC | *B. cereus* | JQ039142.1 | No significant similarity found | | | |
| HblD | *B. cereus* | JQ039158.1 | No significant similarity found | | | |
| NheA, B, C | *B. cereus* | DQ885236.1 | 131 | 5% | 2.00E-31 | 70% |
|  |  |  | 428 | 15% | 4.00E-21 | 70% |
| NheA, B, C | *B. weihenstephanensis* | DQ153260.1 | No significant similarity found | | | |
| NheA | *B. cereus* | FN825684.1 | No significant similarity found | | | |
| Entertoxin | *B. thuringiensis* | EU925144.1 | No significant similarity found | | | |
| entFM | *B. cereus* | AY789084.1 | 103 | 17% | 6.00E-10 | 76% |
| cytK | *B. mycoides* | AY871809.1 | No significant similarity found | | | |
| cytK | *B. licheniformis* | KM657965.1 | No significant similarity found | | | |
| cytK | *B. cereus* | DQ019311.1 | No significant similarity found | | | |
