# Supplementary material for: Safety Assessment of Bacillus subtilis MB40 for Use in Foods and Dietary Supplements
Source: Nutrients. 2021 Feb 25;13(3):733. doi: 10.3390/nu13030733 (PMC7996492; doi:10.3390/nu13030733)
Supplement: Supplementary file 1 [file nutrients-13-00733-s001.zip › MB40 Safety and Tolerability Table S3 BLASTx.docx]

Table S3: Summary of BLASTx screening results for *Bacillus* toxins in MB40

| **Protein** | **Organism** | **GeneBank** | **Accession length** | **% Identical** | **E-value** |
| --- | --- | --- | --- | --- | --- |
| gatA | *B. subtilis* | QGU25606.1 | 485 | 99.8% | 0.0 |
| gyrA | *B. cereus* | AAP07116.1 | 824 | 79.4% | 0.0 |
| metG | *B. cereus* | AIY75532.1 | 660 | 74.9% | 0.0 |
| SpoIVA | *B. cereus* | ACK59614.1 | 492 | 88.0% | 0.0 |
| SpoVT | *B. cereus* | QBZ23122.1 | 178 | 77.0% | 1E-92 |
| HblA | *Multispecies Bacillus* | WP_000976162.1 | 466 | No significant similarity found | |
| HblA | *B. cereus* | QLF02337.1 | 466 | No significant similarity found | |
| HblC | *B. cereus* | CAB69795.1 | 447 | No significant similarity found | |
| HblD | *B. cereus* | CAB69796.1 | 406 | No significant similarity found | |
| NheA | *B. mycoides* | AAZ82477.1 | 386 | No significant similarity found | |
| NheA | *B. cereus* | AAZ82471.1 | 386 | No significant similarity found | |
| NheB | *B. cereus* | AAZ82472.1 | 402 | No significant similarity found | |
| NheC | *B. cereus* | AAZ82473.1 | 359 | No significant similarity found | |
| Enterotoxin | *B. thuringiensis* | OTW41335.1 | 359 | No significant similarity found | |
| Enterotoxin B | *B. licheniformis* | AIS75095.1 | 105 | No significant similarity found | |
| Enterotoxin | *B. anthracis* | QBJ6760.1 | 359 | No significant similarity found | |
| entFM | *B. wiedmannii* | SCL92408.1 | 426 | 52.2% | 2E-30 |
| entFM | *B. mycoides* | AAW82450.1 | 285 | 53.7% | 6E-12 |
| entFM | *B. cereus* | AAX14641.1 | 426 | 52.2% | 3E-30 |
| cytK | *B. mycoides* | AAW56196.1 | 237 | No significant similarity found | |
| cytK | *B. licheniformis* | AIS75096.1 | 127 | No significant similarity found | |
| cytK | *B. cereus* | AKJ86800.1 | 253 | No significant similarity found | |
