# Supplementary material for: Safety Assessment of Bacillus subtilis MB40 for Use in Foods and Dietary Supplements
Source: Nutrients. 2021 Feb 25;13(3):733. doi: 10.3390/nu13030733 (PMC7996492; doi:10.3390/nu13030733)
Supplement: Supplementary file 1 [file nutrients-13-00733-s001.zip › MB40 Safety and Tolerability Table S4 and S5 body weights-210130.docx]

Table S4: Summary of animal body weights (males) from short-term toxicology study

|  | | | | | | |
| --- | --- | --- | --- | --- | --- | --- |
|  |  | Group: | 0 MG/KG/DAY | 500 MG/KG/DAY | 1000 MG/KG/DAY | 2000 MG/KG/DAY |
| Day | -9 |  |  |  |  |  |
|  |  | Mean | 148 | 149 | 149 | 148 |
|  |  | % Difference | N/A | 0.7 | 0.7 | 0 |
|  |  | S.D. | 11.1 | 11.1 | 8.9 | 10.5 |
|  |  | S.E. | 3.5 | 3.5 | 2.8 | 3.3 |
|  |  | N | 10 | 10 | 10 | 10 |
|  |  |  |  |  |  |  |
|  | -2 |  |  |  |  |  |
|  |  | Mean | 222 | 223 | 223 | 223 |
|  |  | % Difference | N/A | 0.5 | 0.5 | 0.5 |
|  |  | S.D. | 13.5 | 14.3 | 15.0 | 15.1 |
|  |  | S.E. | 4.3 | 4.5 | 4.8 | 4.8 |
|  |  | N | 10 | 10 | 10 | 10 |
|  |  |  |  |  |  |  |
|  | 0 |  |  |  |  |  |
|  |  | Mean | 232 | 236 | 234 | 230 |
|  |  | % Difference | N/A | 1.7 | 0.9 | -0.9 |
|  |  | S.D. | 15.6 | 15.1 | 16.4 | 15.8 |
|  |  | S.E. | 4.9 | 4.8 | 5.2 | 5.0 |
|  |  | N | 10 | 10 | 10 | 10 |
|  |  |  |  |  |  |  |
|  | 7 |  |  |  |  |  |
|  |  | Mean | 284 | 293 | 288 | 282 |
|  |  | % Difference | N/A | 3.2 | 1.4 | -0.7 |
|  |  | S.D. | 24.7 | 18.8 | 21.6 | 17.9 |
|  |  | S.E. | 7.8 | 5.9 | 6.8 | 5.6 |
|  |  | N | 10 | 10 | 10 | 10 |
|  |  |  |  |  |  |  |
|  | 13 |  |  |  |  |  |
|  |  | Mean | 324 | 337 | 329 | 324 |
|  |  | % Difference | N/A | 4.0 | 1.5 | 0.0 |
|  |  | S.D. | 28.8 | 22.0 | 25.2 | 20.7 |
|  |  | S.E. | 9.1 | 7.0 | 8.0 | 6.5 |
|  |  | N | 10 | 10 | 10 | 10 |

| Table S5: Summary of animal body weights (female) from short-term toxicology study | | | | | | |
| --- | --- | --- | --- | --- | --- | --- |
|  |  | Group: | 0  MG/KG/DAY | 500 MG/KG/DAY | 1000 MG/KG/DAY | 2000 MG/KG/DAY |
| Day | -10 |  |  |  |  |  |
|  |  | Mean | 136 | 135 | 136 | 135 |
|  |  | % Difference | N/A | -0.7 | 0.0 | -0.7 |
|  |  | S.D. | 7.1 | 9.5 | 7.5 | 8.4 |
|  |  | S.E. | 2.2 | 3.0 | 2.4 | 2.6 |
|  |  | N | 10 | 10 | 10 | 10 |
|  |  |  |  |  |  |  |
|  | -3 |  |  |  |  |  |
|  |  | Mean | 172 | 172 | 172 | 171 |
|  |  | % Difference | N/A | 0.0 | 0.0 | -0.6 |
|  |  | S.D. | 10.1 | 9.2 | 11.1 | 11.1 |
|  |  | S.E. | 3.2 | 2.9 | 3.5 | 3.5 |
|  |  | N | 10 | 10 | 10 | 10 |
|  |  |  |  |  |  |  |
|  | 0 |  |  |  |  |  |
|  |  | Mean | 183 | 185 | 183 | 185 |
|  |  | % Difference | N/A | 1.1 | 0.0 | 1.1 |
|  |  | S.D. | 11.9 | 12.5 | 11.1 | 12.6 |
|  |  | S.E. | 3.8 | 4.0 | 3.5 | 4.0 |
|  |  | N | 10 | 10 | 10 | 10 |
|  |  |  |  |  |  |  |
|  | 7 |  |  |  |  |  |
|  |  | Mean | 207 | 211 | 210 | 211 |
|  |  | % Difference | N/A | 1.9 | 1.4 | 1.9 |
|  |  | S.D. | 15.5 | 13.9 | 10.8 | 15.4 |
|  |  | S.E. | 4.9 | 4.4 | 3.4 | 4.9 |
|  |  | N | 10 | 10 | 10 | 10 |
|  |  |  |  |  |  |  |
|  | 13 |  |  |  |  |  |
|  |  | Mean | 222 | 224 | 226 | 225 |
|  |  | % Difference | N/A | 0.9 | 1.8 | 1.4 |
|  |  | S.D. | 18.7 | 15.4 | 11.5 | 18.9 |
|  |  | S.E. | 5.9 | 4.9 | 3.6 | 6.0 |
|  |  | N | 10 | 10 | 10 | 10 |
